# Muscle-specific deletion of BDK amplifies loss of myofibrillar protein during protein undernutrition

**DOI:** 10.1038/srep39825

**Published:** 2017-01-04

**Authors:** Takuya Ishikawa, Yasuyuki Kitaura, Yoshihiro Kadota, Yukako Morishita, Miki Ota, Fumiya Yamanaka, Minjun Xu, Masahito Ikawa, Naokazu Inoue, Fuminori Kawano, Naoya Nakai, Taro Murakami, Shinji Miura, Yukino Hatazawa, Yasutomi Kamei, Yoshiharu Shimomura

**Affiliations:** 1 Laboratory of Nutritional Biochemistry, Department of Applied Molecular Biosciences, Graduate School of Bioagricultural Sciences, Nagoya University, Nagoya, Japan; 2 Animal Resource Center for Infectious Diseases, Research Institute for Microbial Diseases, Osaka University, Suita, Osaka, Japan; 3 Department of Experimental Genome Research, Research Institute for Microbial Diseases, Osaka University, Suita, Osaka, Japan; 4 Graduate School of Health Sciences, Matsumoto University, Matsumoto, Nagano, Japan; 5 School of Human Cultures, University of Shiga Prefecture, Hikone, Shiga, Japan; 6 Department of Nutrition, Shigakkan University, Obu, Aichi, Japan; 7 Graduate School of Nutritional and Environmental Sciences, University of Shizuoka, Shizuoka, Japan; 8 Graduate School of Environmental and Life Science, Kyoto Prefectural University, Kyoto, Japan

## Abstract

Branched-chain amino acids (BCAAs) are essential amino acids for mammals and play key roles in the regulation of protein metabolism. However, the effect of BCAA deficiency on protein metabolism in skeletal muscle *in vivo* remains unclear. Here we generated mice with lower BCAA concentrations by specifically accelerating BCAA catabolism in skeletal muscle and heart (BDK-mKO mice). The mice appeared to be healthy without any obvious defects when fed a protein-rich diet; however, bolus ingestion of BCAAs showed that mTORC1 sensitivity in skeletal muscle was enhanced in BDK-mKO mice compared to the corresponding control mice. When these mice were fed a low protein diet, the concentration of myofibrillar protein was significantly decreased (but not soluble protein) and mTORC1 activity was reduced without significant change in autophagy. BCAA supplementation in drinking water attenuated the decreases in myofibrillar protein levels and mTORC1 activity. These results suggest that BCAAs are essential for maintaining myofibrillar proteins during protein undernutrition by keeping mTORC1 activity rather than by inhibiting autophagy and translation. This is the first report to reveal the importance of BCAAs for protein metabolism of skeletal muscle *in vivo*.

Muscle proteins are maintained by the balance between protein synthesis and degradation, which are coordinately regulated by nutrients and hormones^1^. In conditions dominated by protein degradation, such as weightlessness and glucocorticoid treatment, muscle proteins (especially myofibrillar proteins) are decreased, which is typically observed in muscle atrophy and myofibrillar myopathy. On the other hand, decreased protein degradation or enhanced protein synthesis can induce muscle hypertrophy. Nutrients and hormones stimulate protein synthesis through mRNA translation. Activation of protein synthesis is positively correlated with the phosphorylation of eukaryotic initiation factor (eIF) 4E-binding protein 1 (4E-BP1) and ribosomal protein S6 kinase (S6K1), which are involved in the initiation of mRNA translation^2^. 4E-BP1 and S6K1 are phosphorylated by mammalian target of rapamycin complex 1 (mTORC1) which regulates the binding of eIF4E to the mRNA 5’cap through phosphorylation of 4E-BP1 and stimulates protein synthesis through the phosphorylation of S6K1^3^,^4^,^5^,^6^. To date, it has been shown that two signaling pathways (growth hormone receptor pathway and amino acid sensing pathway) coordinately activate mTORC1^7^,^8^. Growth hormones such as insulin can initiate the activation of the Akt-Tsc-Rheb cascade through their receptors^7^. The amino acid sensing pathway has been elucidated recently to involve the Sestrin/CASTOR-GATOR-Rag cascade^8^,^9^,^10^. mTORC1 also regulates protein degradation by inhibiting autophagy, which plays a role in the non-selective degradation of subcellular organelles^11^,^12^. On the other hand, protein synthesis is attenuated by eukaryotic initiation factor 2α (eIF2α) which is phosphorylated (activated) by general control nonderepressible 2 (GCN2) in response to amino acid deprivation^13^.

Leucine, isoleucine and valine have branched structures in their side chains, and the so-called branched-chain amino acids (BCAAs) are essential amino acids for mammals. Recent studies have demonstrated that BCAAs play key roles in the regulation of not only protein metabolism but also glucose metabolism^14^,^15^. BCAAs, especially leucine, have been reported to improve protein synthesis and to suppress protein degradation in muscle^16^,^17^,^18^,^19^,^20^,^21^,^22^,^23^. BCAAs are catalyzed in mitochondria in 7 to 10 steps to eventually become acetyl-CoA or succinyl-CoA, a citric acid cycle intermediate^24^,^25^,^26^. The first two steps are common to the three BCAAs. The first step is reversible transamination by BCAA aminotransferase (BCAT). The second step is irreversible decarboxylation by the branched-chain α-keto acid dehydrogenase (BCKDH) complex, and this step is thought to be a rate-limiting step in BCAA catabolism. The BCKDH complex is inactivated by phosphorylation for which BCKDH kinase (BDK) is responsible regulator^27^,^28^.

As stated above, BCAAs, especially leucine, are involved in the improvement and maintenance of muscles. Shimizu *et al*. recently showed that BCAAs improve muscle atrophy in glucocorticoid-treated mice in which upregulated REDD1 and KLF15 induced ubiquitin ligases and BCAT2, resulting in decreased muscle protein and BCAA concentrations^29^. However, there is no direct evidence showing the effect of lowered BCAAs on protein metabolism *in vivo*. In the present study, we generated mice with defective muscle-specific BDK to chronically accelerate BCAA catabolism, resulting in lower BCAA concentrations in muscles, to directly investigate the effect of lowered BCAAs in muscles. We found that muscle-specific BDK knockout (BDK-mKO) mice are healthy without any obvious defects when fed a regular protein diet (RD); however, mice fed a low protein diet (LPD) exhibited a significant decrease in the concentration of myofibrillar, but not soluble protein, in skeletal muscles. The phosphorylation levels of S6K1, 4E-BP1 were reduced in mice fed the LPD. Supplementation of BCAAs ameliorated the defect in maintaining myofibrillar proteins in BDK-mKO mice fed the LPD, suggesting that maintaining BCAA concentrations in skeletal muscle is essential for preserving myofibrillar protein concentrations.

## Results

### Characterization of BDK-mKO mice

**Figure 1:**
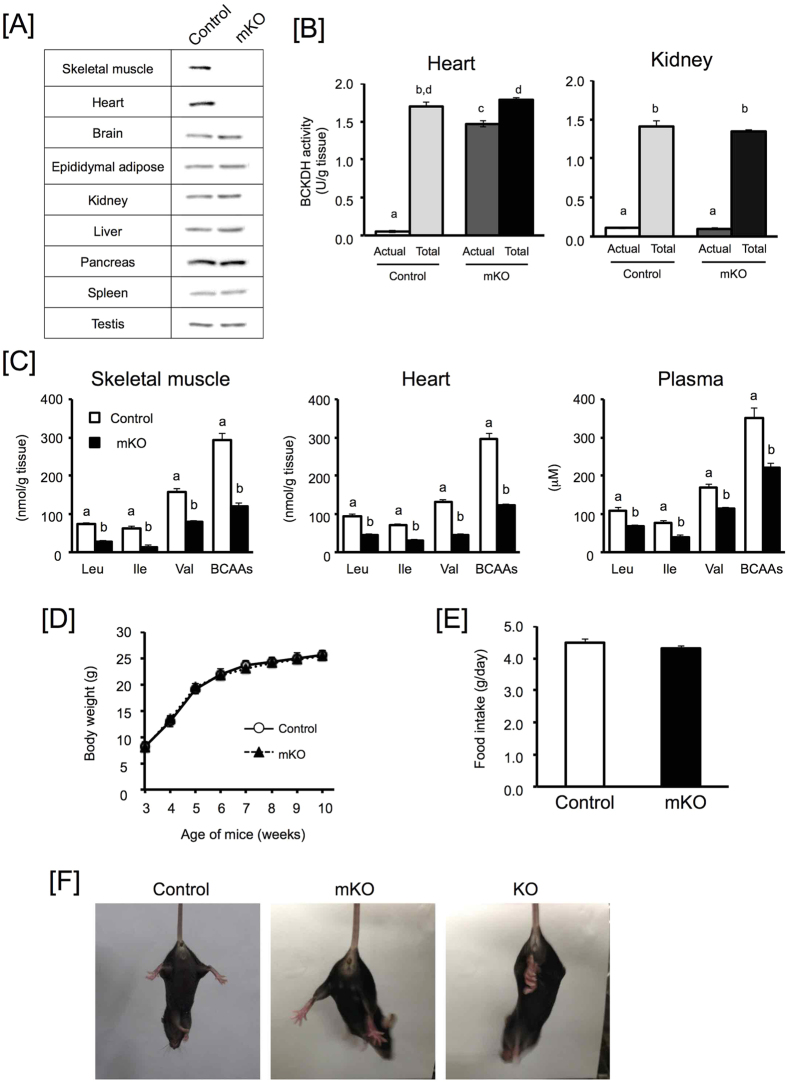
Characterization of BDK-mKO mice. BDK protein expression in various tissues (**A)**, Actual and total BCKDH activity in heart and kidney (**B)**, BCAA concentrations in skeletal muscle, heart and plasma (**C)** in 8 h-fasted control and BDK-mKO mice fed CE-2 (25% protein). (**D)** Growth curves and (**E)** food intake in coAbstntrol and BDK-mKO mice fed CE-2. (**F)** BDK-mKO mice not showing hind limb clasping as seen in BDK-KO mice. Values are presented as mean ± SE (n = 10–12 except heart BCAA concentrations in (**C**) (n = 4–6, two heart sample were pooled and determined as a single sample)). Means without common letters are significantly different (P < 0.05 by Student’s t-test).

We used Cre-*lox*P-mediated gene targeting to specifically delete the *BDK* gene in skeletal muscle and cardiac myocytes. As shown in Supplementary Fig. S1A, mice harboring *lox*P sites flanking exons 9 to 12 of *BDK* (*BDK* floxed mice) were generated and mated to Cre-transgenic mice expressing Cre recombinase under the control of the muscle creatine kinase promoter (Ckmm-Cre;^30^). The *BDK* floxed mice expressing Mmck-Cre were used as the BDK-mKO mice, whereas control mice were *BDK*-floxed mice lacking Cre expression. BDK expression in skeletal muscle and heart, but not in other tissues, was completely abolished in BDK-mKO mice (Fig. 1A). To investigate the effect of BDK deletion in skeletal muscle and heart, BCKDH complex activity and amino acid concentrations were analyzed. BDK phosphorylates and inactivates the BCKDH complex, which is a rate-limiting enzyme in BCAA catabolism^27^,^28^. The actual activity of the BCKDH complex was dramatically lower than the total activity in heart and kidney from control mice (Fig. 1B), indicating that BCKDH complex is almost completely suppressed in these tissues. However, the actual activity of heart, but not kidney, was greater in BDK-mKO mice than control mice (Fig. 1B). These results suggest that BCKDH is constitutively activated specifically in muscle tissues. BCAAs were significantly lower in skeletal muscle and heart (40% of control mouse) as well as in plasma (60% of control mouse) of BDK-mKO mice compared to control mice (Fig. 1C). However other amino acids were not significantly changed and only alanine and glutamine concentrations were slightly higher in plasma of BDK-mKO mice (Table 1). These results indicate that constitutively activated BCAA catabolism in skeletal muscle and heart induces lowered BCAA concentrations not only in skeletal muscle and heart, but also in plasma. A previous report has shown that plasma BCAA concentrations in mice with whole body BDK deficiency (BDK-KO mice) was decreased to 50% of control mice^31^, suggesting that skeletal muscle and heart mainly modulate circulating BCAA homeostasis.

It has also been reported that the BDK-KO mice showed lower body weight than control wild-type mice, and neurological abnormalities characterized by hind limb clasping^31^. We also generated mice with whole body BDK-deletion (BDK-KO mice) using the BDK floxed mice with a *FRT*-flanked LacZ/neo cassette (BDK floxed (Neo+)) (Supplementary Fig. S1A). The BDK-KO mice showed low body weight (Supplementary Fig. S1B) and hind limb clinching (Fig. 1F), as previously reported; however the BDK-mKO mice showed normal growth, food intake, and tissue weights without any obvious neurological defects (Fig. 1D–F, Table 2), even though the decreased plasma BCAA concentrations of the BDK-mKO mice was similar to the levels observed in the BDK-KO mice. These results suggest that the observed impaired growth rate and neurological defects are due to decreased BCAA concentration in tissues other than skeletal muscle and heart, and not attributable to decreased plasma BCAA concentrations. We also analyzed muscle fiber types (Supplementary Fig. S2), skeletal muscle protein concentrations (Supplementary Fig. S3A), mitochondria enzyme activities in skeletal muscle and heart (Supplementary Fig. S3B), and gene expression pattern (Supplementary Table S1 and Fig. S4); however, there were no obvious differences between control and BDK-mKO mice.

### The effects of bolus ingestion of BCAAs on mTORC1 activity in BDK-mKO mice

**Figure 2:**
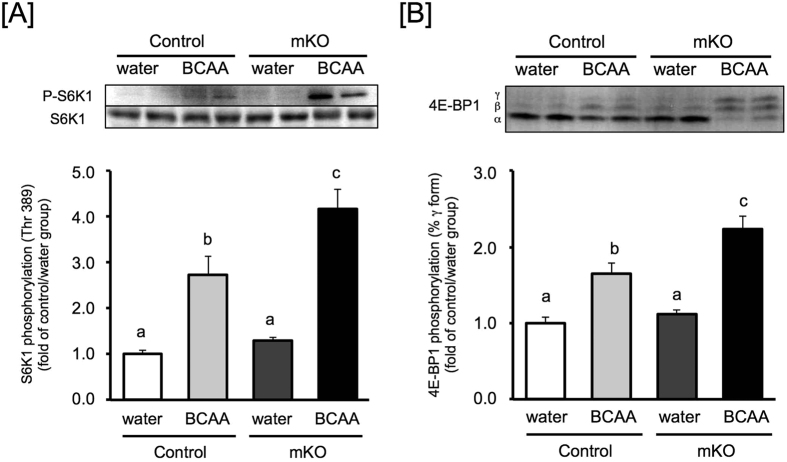
Effect of bolus BCAA administration on activity of the mTORC1 pathway in skeletal muscle of control and BDK-mKO mice. The phosphorylation states of S6K1 (**A)** and 4E-BP1 (**B)** in 12 h-fasted skeletal muscle of control and BDK-mKO mice fed RD (20% protein) at 1 h after oral administration of water or BCAA solution. Phosphorylation of 4E-BP1 was expressed as a percentage of the γ-form to total 4E-BP1 (α + β + γ-form). The data are presented relative to the mean values of the control/water group. Typical images of western blots are shown above each bar. Values are presented as mean ± SE (n = 4–6). Means without common letters are significantly different (P < 0.05 by the Tukey-Kramer test).

Oral BCAA, especially leucine administration, has been shown to activate mTORC1 in skeletal muscle^32^,^33^. Thus, we examined the effect of a single bolus ingestion of BCAAs on the mTORC1 pathway in skeletal muscle of BDK-mKO mice. BCAA concentrations in plasma and skeletal muscle of the control mice were dramatically increased at 1 h after the ingestion of BCAAs, and these concentrations were almost 8- and 6-fold higher than those of water-treated control mice, respectively (Supplementary Table S2). In the BDK-mKO mice, the BCAA concentrations were dramatically increased, similar to those in control mice, with concentrations almost 6- or 10-fold higher in plasma and 5- or 8-fold higher in skeletal muscle than water-treated control or BDK-mKO mice, respectively (Supplementary Table S2). The BCAA concentrations in both plasma and skeletal muscle of BDK-mKO mice were around 80% of those of control mice when administered BCAAs (Supplementary Table S2), suggesting that administrated BCAAs are quickly catabolized in BDK-mKO mice. To assess mTORC1 activation by BCAA ingestion, the phosphorylation states of the components in the mTORC1 pathway were expressed as a ratio relative to that of the water-treated control group. In the BCAA-treated groups, S6K1 phosphorylation in the BDK-mKO mice group was increased (3.8-fold) to a greater degree than in control mice (2.5-fold) (Fig. 2A). The tendency of the phosphorylation state of 4E-BP1 was similar to that of S6K1 following ingestion of BCAAs (Fig. 2B). These results suggest that chronically decreased BCAA concentrations in skeletal muscle elevate the sensitivity of the mTORC1 reaction to BCAAs.

### The effects of low protein diet on muscle protein of BDK-mKO mice

**Figure 3:**
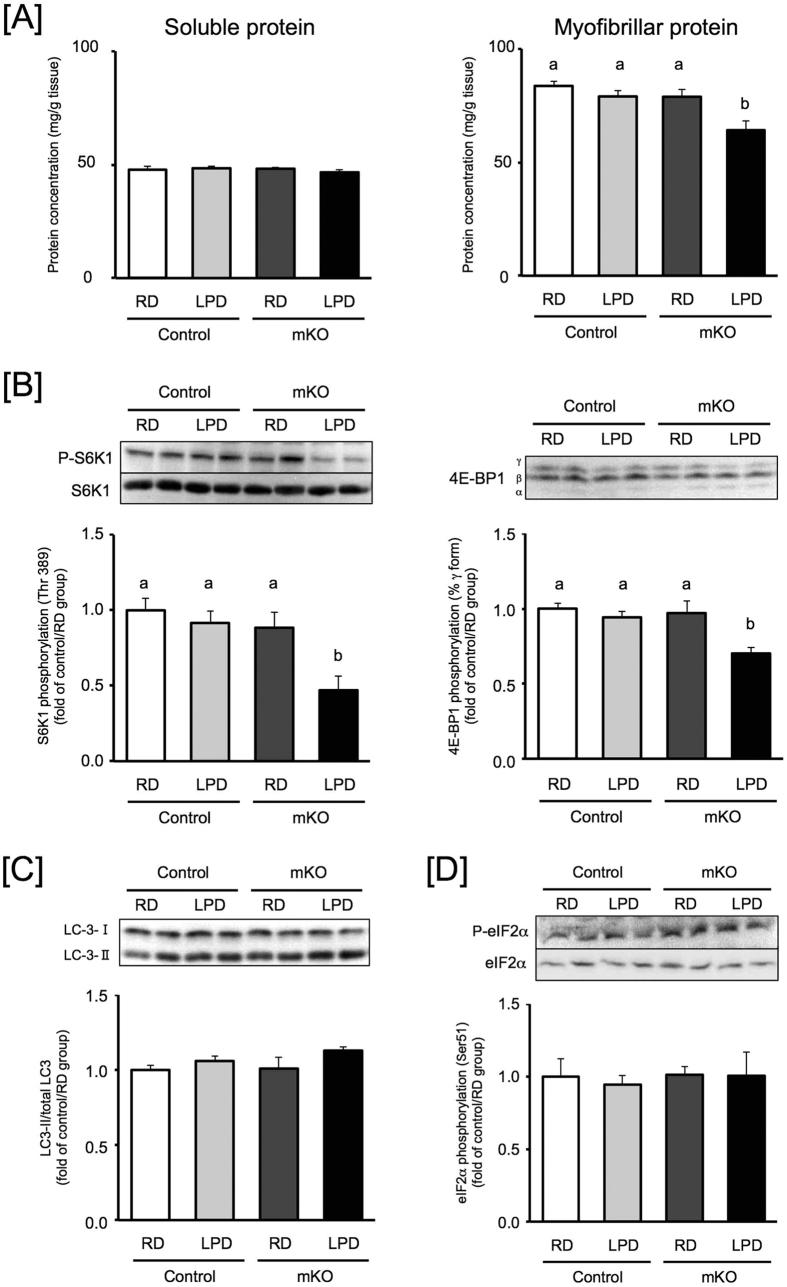
Skeletal muscle protein concentrations and mTORC1 activity in control and BDK-mKO mice fed RD or LPD. (**A)** Soluble and myofibrillar protein concentrations in skeletal muscle of 2 h-fasted control and BDK-mKO mice fed RD (20% protein) or LPD (8% protein) for 12 weeks. The protein concentrations are expressed as mg/g of tissue weight for skeletal muscle (gastrocnemius and plantaris muscle). The activity of mTORC1 (**B)**, conversion of LC3 (**C)** and the activity of eIF2a (**D)** in skeletal muscle of 2 h-fasted control and BDK-mKO mice fed RD or LPD for 12 weeks. The phosphorylated levels of S6K1 and 4E-BP1 are expressed as in Fig. 2. The converted level of LC3 is expressed as a percentage of the LC3-II to total LC3 (I+II). The data are presented relative to the mean values of the control/RD group. Typical images of western blots are shown above each bar. Values are presented as mean ± SE (n = 4–7). Means without common letters are significantly different (P < 0.05 by the Tukey-Kramer test).

We found that there were no obvious defects in skeletal muscle of BDK-mKO mice when fed RD with 20% protein content (Supplementary Table S6). In comparison, we analyzed the effect of LPD with 8% protein content (Supplementary Table S6) on BDK-mKO mice by feeding either RD or LPD to control and BDK-mKO mice for 12 weeks. The BDK-mKO mice fed LPD exhibited slightly decreased body weight compared to the other groups of mice (Supplementary Fig. S5A), while food intake of mice fed LPD was significantly greater than those of mice fed RD (Supplementary Fig. S5B). The concentrations of BCAAs in control mice fed LPD, and BDK-mKO mice fed RD or LPD were decreased to approximately 75%, 70% and 50% in plasma (Supplementary Fig. S5C), and 80%, 60% and 50% in skeletal muscle (Supplementary Fig. S5D) compared to that of control mice fed RD, respectively. However other amino acids were not significantly decreased in BDK-mKO mice fed LPD compared to other mice (Supplementary Table S5). The weights of skeletal muscle (relative to body weight) were not significantly changed among the four groups (Supplementary Table S3). We fractionated the skeletal muscles to soluble and myofibrillar proteins. As shown in Fig. 3A, soluble proteins were not changed among the four groups, but myofibrillar proteins were significantly decreased in BDK-mKO mice fed LPD compared to other groups. We also analyzed the activity of mTORC1 and the conversion of LC3, a marker for autophagosome. BDK-mKO mice fed LPD showed lower levels of phosphorylated S6K1 and 4E-BP1 than the other groups (Fig. 3B); however, the levels of converted LC3-II (membrane-bound form) from LC3-I (soluble form) were not changed among the four groups (Fig. 3C). Previous reports have showed that phosphorylated eIF2α was increased in brain of BDK-KO mice^31^, whereas that response was diminished by GCN2 deficiency^34^. To determine whether in skeletal muscle, BCAAs deficiency inhibits protein synthesis through the phosphorylation of eIF2α which triggers a reduction in protein synthesis, we also analyzed the phosphorylation states of eIF2α. As shown in Fig. 3D, there were no differences in the phosphorylated levels of eIF2α among these groups. In addition, although the phenotype described above was found in LPD feeding for 12-weeks, BDK-mKO mice fed LPD for 4-weeks did not show significant decrease in myofibrilar protein concentration and mTORC1 activity (Supplementary Fig. S6).

**Figure 4:**
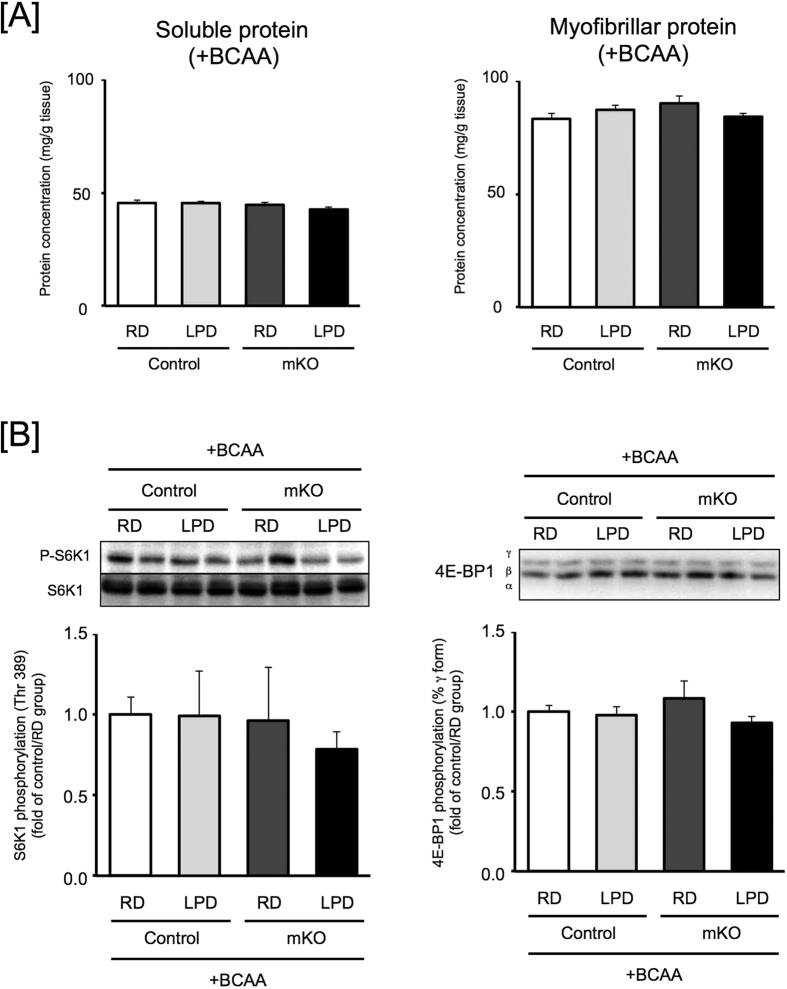
Skeletal muscle protein concentration and mTORC1 activity in control and BDK-mKO mice fed RD or LPD with BCAA supplemented water. (**A)** The soluble and myofibrillar protein concentration in skeletal muscle of 2 h-fasted control and BDK-mKO mice fed RD (20% protein) or LPD (8% protein) for 12 weeks with BCAA supplemented water. The protein concentrations are expressed as in Fig. 3. (**B**) The activity of mTORC1 in skeletal muscle of 2 h-fasted control and BDK-mKO mice fed RD or LPD for 12 weeks with BCAA supplemented water. The phosphorylation states of S6K1 and 4E-BP1 were determined as in Fig. 2. Typical images of western blots are shown above each bar. Values are presented as mean ± SE (n = 5–8). Means without common letters are significantly different (P < 0.05 by the Tukey-Kramer test).

Next, to investigate whether the decreased myofibrillar proteins were attributed to lowered BCAA concentrations in skeletal muscle, we examined the effects of BCAA supplementation (Leu: Val: Ile = 2: 1: 1; 3% in tap water; BCAA-water) on the muscle protein content. Growth curves were not changed among the groups of mice (Supplementary Fig. S6A). The LPD-fed mice consumed more BCAA-water than the RD-fed mice (Supplementary Fig. S5B); however, food intake did not differ among the four groups when mice were given BCAA-water (Supplementary Fig. S5C). The food intake of LPD-fed mice was decreased by BCAA-water (Supplementary Figs S4C and S5C), suggesting that BCAAs regulate food intake driven by compensatory feeding for protein. The concentrations of BCAAs in plasma and skeletal muscles of control and BDK-mKO mice were increased by supplementation with BCAA-water (Supplementary Figs S6D and S6E). The weights of skeletal muscle were not different among the four groups (Supplementary Table S4). Although the concentration of myofibrillar proteins and the activity of the mTORC1 pathway were decreased in the LPD-fed BDK-mKO mice without BCAA supplementation (Fig. 3A,B), the LPD-fed mice given BCAA-water did not show significant differences in myofibrillar proteins and mTORC1 activity among the groups (Fig. 4A,B). These data suggest that supplementation of BCAAs improved the maintenance of muscle myofibrillar proteins in LPD-fed mice by increasing mTORC1 activity.

## Discussion

Mice lacking BDK throughout the body (BDK-KO mice) exhibited neurological defects, and lower body weights compared to mice with normal BDK expression, which were ameliorated by supplementation of BCAAs or high-protein diet^31^,^35^. The BDK-KO mice were approximately 15% smaller than wild-type mice, and several tissue weights, such as brain, muscle and adipose tissue, were also reduced^31^. The muscle-specific BDK-deleted (BDK-mKO) mice generated in this report exhibited a normal growth rate, tissue weights and muscle fibers, suggesting that preserving BCAA concentrations in tissues other than skeletal muscle and heart (probably brain) is important for maintaining body and tissue weights. BCAA concentrations were decreased in the skeletal muscle and heart (40% of control mice), as well as in the plasma (60% of control mice) of BDK-mKO mice, suggesting that skeletal muscle and heart play a key role in regulating plasma BCAA concentrations. Herman *et al*. suggested that circulating BCAA levels are also modulated by adipose tissue BCAA catabolism^36^. It is interesting to investigate plasma BCAA levels of the mice with constitutively activated BCAA catabolism specifically in adipose tissue using the BDK floxed mouse generated in this study. Even though both BDK-KO and mKO mice showed significantly lowered plasma BCAA levels, the neurological defects were found only in BDK-KO mice. The BCAA catabolism (especially the first step by BCAT) plays an important role to replenish glutamate in astrocytes as well as to avoid high concentrations of glutamate in neurons by re-aminating branched-chain α-ketoacids (BCKA)^37^. The abnormalities in BDK-KO mice might be induced, not by the decreased circulating BCAA concentrations, but by perturbed “Glutamate-BCAAs cycle”.

It is well known that BCAAs, especially leucine, play a key role in promoting protein synthesis and inhibiting protein degradation through mTORC1. *In vivo* experiments have shown that the oral BCAA administration activates mTORC1 in skeletal muscle^32^,^33^. Surprisingly, mTORC1 activity was significantly higher in BDK-mKO mice than control mice (Fig. 2). This result indicates that lowered BCAA concentrations due to accelerated catabolism can increase the sensitivity of skeletal muscle protein synthesis to BCAAs, which might be the reason why BDK-mKO mice are able to maintain muscle tissue weight. Myofibrillar proteins are maintained by sequentially repeated feeding and fasting cycles, which induce protein synthesis and degradation, respectively^38^,^39^. In this report, we were surprised to observe that BDK-mKO mice did not exhibit alterations in skeletal muscle tissue weights when fed regular diet (RD) with 20% protein; however, unlike soluble proteins, myofibrillar proteins in skeletal muscle were significantly decreased in BDK-mKO mice compared to control mice when fed a low protein diet (LPD) with 8% protein for 12-weeks. mTORC1 and GCN2 have been shown to control protein metabolism by availabilities of amino acids^5^,^6^,^11^,^12^,^13^. Conversion of the autophagy marker LC3 and phosphorylated level of eIF2 α were not changed even though mTORC1 activity was decreased in BDK-mKO mice fed LPD; suggesting that lowered BCAA concentrations affect protein synthesis through mTORC1, but not soluble protein degradation by autophagy and suppression of translation by GCN2. Different mechanisms might be involved in the stimulation of protein synthesis, and suppression of autophagy and translation through mTORC1 and GCN2. Previous reports have shown a significantly increased phospho-eIF2a in brain of BDK-KO mice^31^ and identified GCN2 as the primary kinase, at least in brain^34^, suggesting that the sensitivities of GCN pathway to BCAAs might be different among the tissues. In addition, the decreased myofibrillar protein and mTORC1 activity were not seen in mice fed LPD for 4-weeks (Supplementary Fig. S7), indicating that chronically maintaining BCAA levels is needed for keeping myofibrillar protein during protein undernutrition. It still remained to elucidate how mice adapt to maintain myofibrillar protein under decreased BCAAs with enough protein intake and how LPD suppresses the adaptation response. The LPD at 8% protein contents used in this report seems likely that the protein level was not low enough to deliver differences in tissue protein content at earlier time point. A greater level of protein restriction using 5% or lower as described in the recent paper^40^ could have amplified differences in protein metabolism and its regulation system between control and BDK-mKO mice. It is also interesting to see what might happen if the mice were fed a diet devoid of BCAA.

As described above, this is the first report to show the direct effect of specifically lowering BCAA concentrations in skeletal muscle and heart. We propose that maintaining BCAA concentrations at normal levels is important for protein synthesis, but not for protein degradation in skeletal muscle *in vivo*, especially in the case of decreased protein intake. Thus, BCAAs could be helpful for people with decreased protein intake, such as the aged, to suppress sarcopenia by promoting protein synthesis. The conditional BDK deficient mice generated in this report are also useful animal models for further investigation to find out the importance of the relationship between BCAA oxidation and protein homeostasis in muscle and other tissues, especially in brain using the right Cre-transgenic mice.

## Methods

### Materials

Antibodies against phospho-S6K1 Thr389 (#9234), 4E-BP1 (#9452) and LC3A/B (#12741), eIF2a (#5324) and phosphor-eIF2a S51 (#3398) were purchased from Cell Signaling Technology (Danvers, MA, USA). Antibodies against S6K1 (sc-230) and horseradish peroxidase (HRP)-conjugated anti-goat immunoglobulin G (IgG) (sc-2768) were obtained from Santa Cruz Biotechnology (Santa Cruz, CA, USA). HRP-conjugated goat anti-rabbit IgG was purchased from Bio-Rad Laboratories (Hercules, CA, USA). Purified diets for mice with 20 kcal% protein (D12450J) as RD and 8 kcal% protein (D06011002) as LPD were prepared by Research diets (New Brunswick, NJ, USA, Supplementary Table S6). An unpurified diet, CE-2 (containing crude protein at 25%) was purchased from CLEA Japan (Tokyo, Japan). All other reagents were of analytical grade and were purchased from Wako (Osaka, Japan) or Sigma Aldrich Japan (Tokyo, Japan).

### Generation of BDK-mKO mice

All animal procedures were approved by the Animal Care Committee of Nagoya University Graduate School of Bioagricultural Sciences, and were performed in accordance with the relevant guidelines. The targeting vector for the *BDK* allele with a *FRT*-flanked LacZ/neo cassette integrated upstream of exon 9 and the floxed exons 9–12 was obtained from European Conditional Mouse Mutagenesis Program (EUCOMM) (Supplementary Fig. S1A). *BDK*-floxed (Neo+) ES cells were generated and implanted in pseudopregnant mothers for creating chimeric mice. The chimeric mice were crossed with C57BL/6 mice to produce heterozygous *BDK*-floxed (Neo+) mice, which were used to generate whole body BDK-KO mice. The *BDK*-floxed (Neo+) mice were mated with FLPe transgenic mice (#RBRC01834, RIKEN BioResource Center)^41^ to remove the *FRT*-flanked LacZ/neo cassette to obtain *BDK* floxed (Neo-) offspring, which were used to produce *BDK*^flox/flox^ mice. The *BDK*^flox/flox^ mice obtained were mated with transgenic mice that have the Cre recombinase gene driven by Ckmm promoter (#006475, Jackson Laboratory)^30^ to generate BDK-mKO (*BDK*^flox/flox^; Ckmm-Cre (+)) mice. *BDK*^flox/flox^; Ckmm-Cre (−) mice were used as control mice. These mice were housed at 23 ± 1 °C with light from 8:00 h to 20:00 h, and free access to water. Male mice were housed individually from 4–5 weeks of age with free access to water and food (CE-2). The mice were deprived of food from 10:00 h to 16:00 h for 8 hours on the final day were sacrificed by exsanguination under isoflurane anesthesia, and blood and tissues were quickly removed, immediately frozen in liquid nitrogen and subsequently stored at −80 °C until use.

### Bolus BCAA administration

Control and BDK-mKO mice were fed RD *ad libitum* until 12 weeks of age. One day before the final day of the experiment, mice were deprived of food from 22:00 h and fasted for 11 h. At 9:00 h on the final day of the experiment, mice were administered BCAAs at 0.9 g/kg body weight (BW) using 3.6% BCAA (Leu:Ile:Val = 2:1:1) suspended in distilled water or the same volume of distilled water as vehicle. Mice were sacrificed 1 h after administration by exsanguination under isoflurane anesthesia, and hindlimb skeletal muscles were quickly removed, immediately frozen in liquid nitrogen, and subsequently then stored at −80 °C until use.

### Dietary manipulation of mice

Control and BDK-mKO mice fed RD at 8 weeks of age were divided into two dietary groups: control mice fed RD or LPD, and mKO mice fed RD or LPD. The mice were individually housed and fed *ad libitum* the appropriate diet and tap water for either 4 weeks or 12 weeks. On the final day of the experiment, the 20 week-old mice were deprived of food from 8:00 h to 10:00 h and then sacrificed by exsanguination under isoflurane anesthesia. Blood was taken from the inferior vena cava with a syringe for preparation of plasma, and hindlimb skeletal muscles (soleus, gastrocnemius, plantaris, quadriceps) were quickly removed, immediately frozen in liquid nitrogen, and then stored at −80 °C until use. For BCAA supplementation, control and mKO mice were prepared and housed using the same procedures as those in the first experiment, except that mice were given a 3% (w/v) solution of BCAAs (Leu:Ile:Val = 2:1:1) in place of tap water during the experiment.

### Measurement of plasma and skeletal muscle amino acid concentrations

Deproteinization of plasma was conducted as follows. Plasma was mixed with an equal volume of 3% (w/v) sulfosalicylic acid, incubated for 15 min at 4 °C, and then the mixture was centrifuged at 15,000 *g* for 15 min to obtain the supernatant. Tissues were powdered at liquid nitrogen temperature, weighed, and extracted with 3% (w/v) sulfosalicylic acid for deproteinization, as described for plasma. The supernatants obtained were filtered through a 0.45 μm pore filter (UFC30HVNB, Merck Millipore, Darmstadt, Germany) and amino acid compositions in the filtrates were measured using the JLC-500/V amino acid autoanalyzer (JEOL, Tokyo, Japan).

### Fractionation and measurement of skeletal muscle proteins

Left hind limb muscles (gastrocnemius and plantaris) were fractioned as described previously^42^ with a few modifications. Frozen muscle was pulverized under the liquid nitrogen temperature, and 10–15 mg of the muscle tissues were weighed and homogenized in 400 μL buffer containing 10 mM Tris/HCl (pH 6.8), 250 mM sucrose, 100 mM KCl, and 5 mM EDTA. Homogenates were centrifuged at 1,000 × *g* for 10 min and the supernatants were collected as the soluble protein fraction for protein determination. The pellets were suspended by vortexing in 400 μL buffer containing 10 mM Tris/HCl (pH 6.8), 175 mM KCl, 2 mM EDTA, and 0.5% (w/v) Triton X-100, centrifuged as above, and the pellets obtained were resuspended with the same buffer. This procedure was repeated a second time. The pellets obtained were resuspended in 400 μL buffer containing 10 mM Tris/HCl (pH 7.0) and 150 mM KCl, and centrifuged as above. Finally, the pellets obtained were resuspended in 500 μL buffer containing 10 mM Tris/HCl (pH 7.4), 100 mM KCl, and 1 mM EDTA and used as the myofiblliar protein fraction. For determination of total protein, pulverized frozen skeletal muscles (10–15 mg) were homogenized in 400 μL buffer containing 10 mM Tris/HCl (pH 6.8), 250 mM sucrose, 100 mM KCl, and 5 mM EDTA. The soluble, myofiblliar, and total protein fractions were diluted with the same volume of 1.5 M NaOH, and protein concentrations in each fraction were determined using the Bicinchoninic acid protein assay kit (Thermo Fisher Scientific, Waltham, MA, USA).

### Western blotting

Frozen tissues were powdered in liquid nitrogen and proteins were extracted with buffer containing 50 mM HEPES (pH 7.4), 1% (w/v) Triton X-100, 1 mM EDTA, 1 mM EGTA, 2 mM Na_3_VO_4_, 100 mM NaF, 50 mM Na_4_P_2_O_7_, 1 mM phenylmethylsulfonyl fluoride (PMSF), 20 μg/mL leupeptin, 5 μg/mL aprotinin, 0.1 mg/mL trypsin inhibitor, 0.1 mM N-tosyl-L-phenylalanine chloromethyl ketone (TPCK). Extracts were centrifuged at 15,000 × *g* for 15 min and supernatants were collected. Protein concentrations were determined using the Bicinchoninic acid protein assay kit. Protein (30–40 μg) was applied to SDS-PAGE and transferred onto a polyvinylidene fluoride (PVDF) membrane (Millipore Corporation, Billerica, MA, USA). Membranes were blocked for 2 h with 5% (w/v) skim milk in Tris-buffered saline (TBS) containing 0.1% (w/v) Tween 20 (TBST) at room temperature. Membranes were consecutively treated as follows: incubation overnight at 4 °C with primary antibodies, washing three times with TBST, incubation for 2 h at room temperature with HRP-conjugated goat anti-rabbit IgG in TBST, and washing two times with TBST and TBS. Target immunoreactive proteins on the membranes were visualized using ECL Western blotting detection reagents (GE Healthcare, Buckinghamshire, UK) and quantified using the AE6962 Light Capture system (ATTO, Tokyo, Japan). The intensities of the bands are expressed relative to the mean values of the control.

### BCKDH complex activity assay

Preparation of heart and kidney extracts and assay of BCKDH complex activity were conducted as reported previously^43^,^44^. One unit of the enzyme activity was defined as the rate of formation of 1 μmol of NADH/min at 30 °C. The actual activity (actually dephosphorylated form *in vivo*) and the total activity (totally dephosphorylated form by treating the tissue extract with lambda protein phosphatase *in vitro*) of BCKDH complex were measured separately^43^.

### Analysis of muscle fiber types

Plantaris and soleus muscles of 12-weeks-old control and BDK-mKO mice fed CE-2 were frozen in liquid nitrogen and the serial cross-sections cut at 10 μm thickness in a cryostat maintained at −20 °C were subjected to immunohistochemical staining for type I or II MHC isoforms and laminin, as well as staining for succinate dehydrogenase (SDH) activity, as described previously^45^,^46^. Fibers stained by either type I or II MHC antibodies were identified as “type I or II fibers”, and those stained by both antibodies were “type I+II fibers”. Oxidative metabolism in the fibers of plantaris was determined by SDH staining. Fibers positively stained by SDH were identified as fast twitch oxidative glycolytic (FOG), whereas negative fibers were described as fast twitch glycolytic (FG), since the plantaris muscle generally contains fewer type I fibers. Fiber cross-sectional area was measured in the laminin (soleus) or SDH (plantaris) stained images using image analysis software (Image J). At least 100 fibers were analyzed in each muscle.

### cDNA microarray analysis

RNA was isolated from skeletal muscle (extensor digitorum longus (EDL) and soleus) of 9- to 10-weeks-old control and BDK-mKO mice fed CE2. Samples from control and BDK-mKO mice (N = 5) were pooled and used. Each sample was labeled with a cyanine 3-CTP using the Low Input Quick Amp Labeling Kit (Agilent Technologies, Santa Clara, CA) and hybridized to the Agilent whole mouse genome microarray (4 × 44 K), which contains 41,534 genes including expressed sequence tags. Signal detection and data analysis were performed as described previously^47^.

### Gene expression analysis of amino acid transporters

Total RNA was extracted from skeletal muscle (gastrocnemius + plantaris + soleus) of 12 h fasted control and BDK-mKO mice fed RD at 12 weeks of age with Isogen II (Nippon Gene, Tokyo, Japan). Complementary DNA (cDNA) was generated using the PrimeScript RT reagent kit (Takara Bio, Ohtsu, Japan). Quantitative reverse transcription polymerase chain reaction (qRT-PCR) was performed to measure mRNA levels of genes encoding the amino acid transporters, as described previously^48^.

### Statistical analysis

All values are expressed as mean ± standard error (SE). Data were analyzed statistically using the Student’s t-test or ANOVA followed by the Tukey-Kramer test. Differences were considered significant at *P* < 0.05.

## Additional Information

**How to cite this article**: Ishikawa, T. *et al*. Muscle-specific deletion of BDK amplifies loss of myofibrillar protein during protein undernutrition. *Sci. Rep.* **7**, 39825; doi: 10.1038/srep39825 (2017).

**Publisher's note:** Springer Nature remains neutral with regard to jurisdictional claims in published maps and institutional affiliations.

## Supplementary Material

Supplementary Information

## Figures and Tables

**Table 1:** Amino acid concentrations in plasma of control and BDK-mKO mice.

Amino acids	Control	BDK-mKO
Alanine	348 ± 16	394 ± 19
Arginine	73 ± 3	69 ± 4
Aspartic acid	7 ± 1	7 ± 2
Glutamic acid	20 ± 3	23 ± 3
Glutamine	502 ± 21	534 ± 19
Glycine	319 ± 14	335 ± 10
Histidine	38 ± 5	46 ± 8
Lysine	194 ± 14	200 ± 9
Methionine	115 ± 7	112 ± 6
Phenylalanine	60 ± 3	58 ± 3
Serine	109 ± 4	110 ± 7
Threonine	113 ± 6	111 ± 5
Tyrosine	77 ± 5	84 ± 6

Comparison of the plasma amino acid concentrations (μM) of 8 h-fasted control and BDK-mKO mice fed CE-2 (25% protein). Values are presented as mean ± SE (n = 10–12).

**Table 2:** Tissue weights of control and BDK-mKO mice.

Tissue	Control	BDK-mKO
Skeletal muscle*	1.08 ± 0.01	1.07 ± 0.02
Heart	0.50 ± 0.02	0.50 ± 0.04
Brain	1.61 ± 0.03	1.61 ± 0.05
Epididymal adipose	0.90 ± 0.06	1.01 ± 0.06
Kidney	1.35 ± 0.06	1.34 ± 0.04
Liver	4.68 ± 0.11	4.59 ± 0.11
Pancreas	0.57 ± 0.04	0.67 ± 0.06
Spleen	0.37 ± 0.05	0.32 ± 0.02
Testis	0.68 ± 0.03	0.66 ± 0.02

Comparison of the weights of various tissues relative to body weight (g/100 g) of 8 h-fasted control and BDK-mKO mice fed CE-2 (25% protein). ^*^Skeletal muscle: Gastrocnemius + Plantaris + Soleus. Values are presented as mean ± SE (n = 10–12).
