# Supplementary material for: Muscle-specific deletion of BDK amplifies loss of myofibrillar protein during protein undernutrition
Source: Sci Rep. 2017 Jan 4;7:39825. doi: 10.1038/srep39825 (PMC5209746; doi:10.1038/srep39825)
Supplement: Supplementary Information [file srep39825-s1.pdf]

Supplementary Information

Muscle-specific deletion of BDK amplifies loss of myofibrillar protein during protein undernutrition

Takuya Ishikawa, Yasuyuki Kitaura, Yoshihiro Kadota, Yukako Morishita, Miki Ota, Fumiya Yamanaka, Minjun Xu, Masato Ikawa, Naokazu Inoue, Fuminori Kawano, Naoya Nakai, Taro Murakami, Shinji Miura, Yukino Hatazawa, Yasutomi Kamei, Yoshiharu Shimomura

[A]

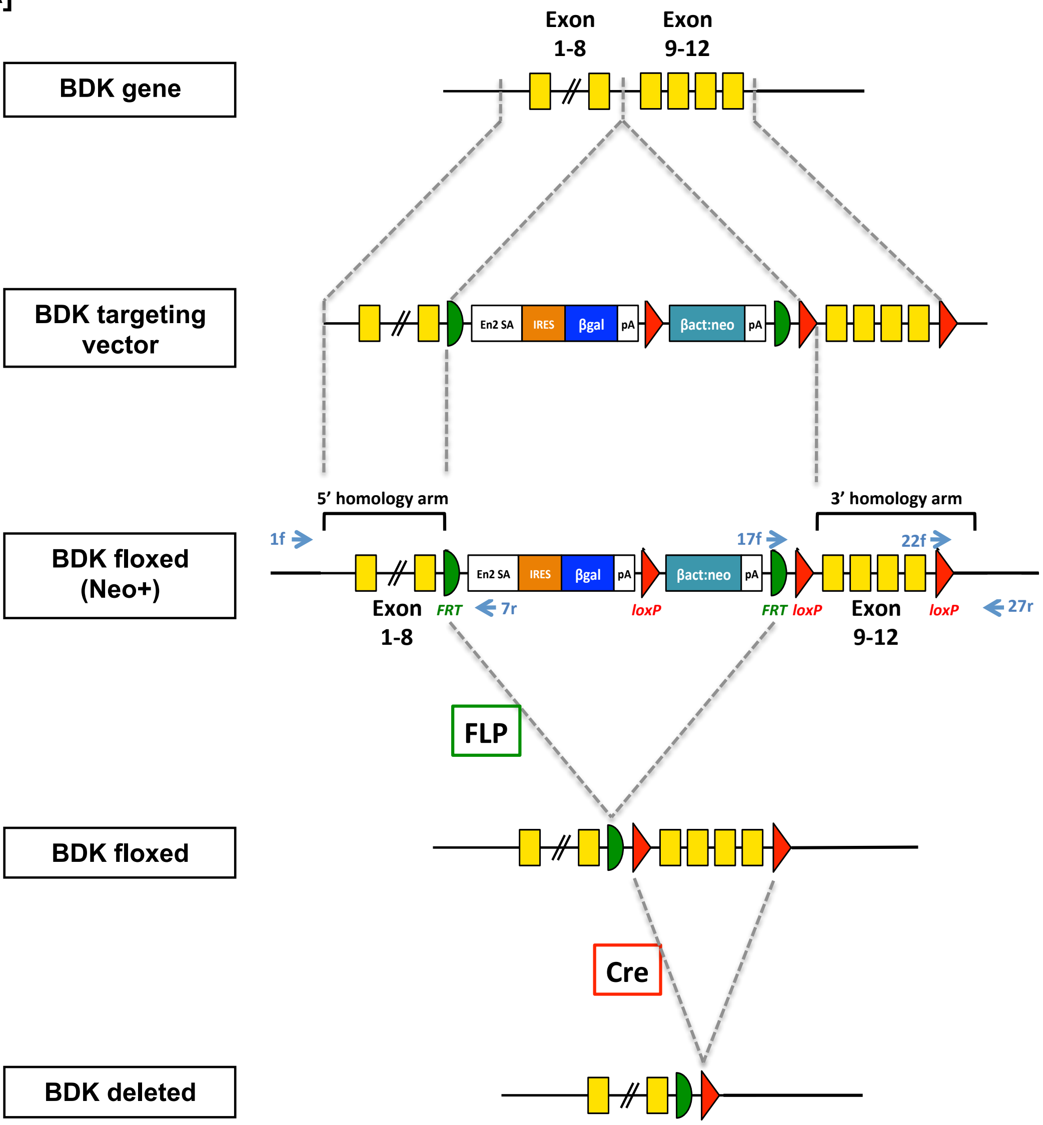

[B]

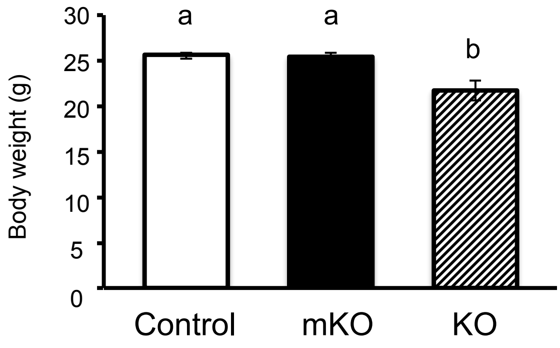

Supplemental Figure S1

Supplemental Figure S1. Generating BDK targeted mice

[A] The targeting vector for the *BDK* allele. Schematic of the targeting vector for *BDK* allele with a *FRT*-flanked βgal/neo cassette integrated upstream of exon 9 and the floxed exons 9-12. En2SA, splicing acceptor site; IRES, internal ribosomal entry site; βgal, β-galactosidase; βact:neo, β-actin-promotor followed by neomycin-resistant gene; pA, poly A sequence. The arrows show primers for PCR for genotyping. **[B]** Body weight of control, BDK-mKO and BDK-KO mice fed CE-2 (25% protein) at 10 weeks. Values are presented as mean ± SE (n=8-12). Means without common letters are significantly different ($P<0.05$ by the Tukey-Kramer test).

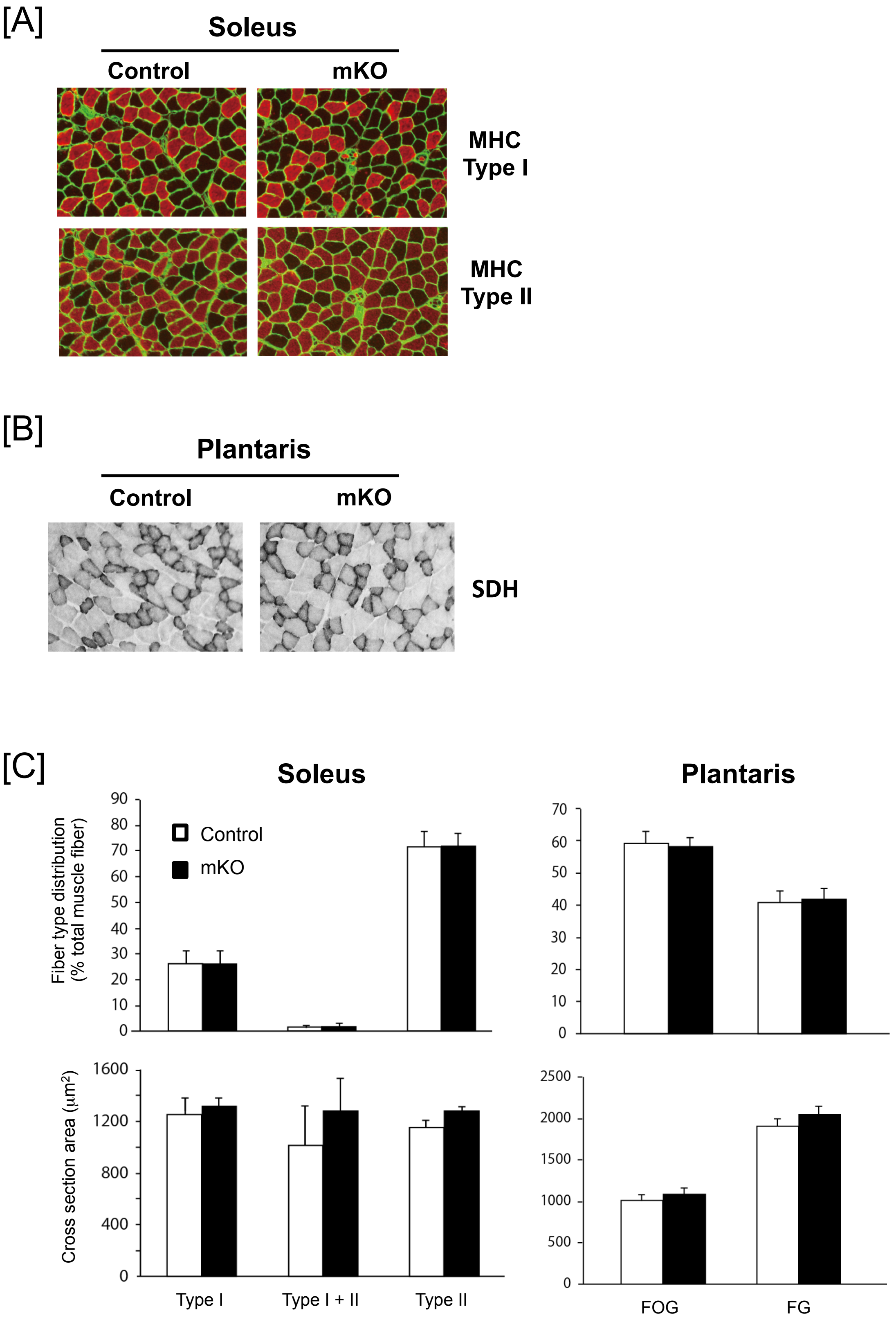

Supplemental Figure S2

Supplemental Figure S2. Muscle fiber characteristics of control and BDK-mKO mice. **[A]** Tissue cross-sections immunohistochemically stained with type I or II myosin heavy chain (MHC) (red) and laminin (green) to outline myofibers of soleus muscle from control and BDK-mKO mice fed CE-2 (25% protein). **[B]** Tissue cross-sections stained for succinate dehydrogenase (SDH) activity (dark gray/black) of plantaris muscle from control and BDK-mKO mice fed CE-2. **[C]** Fiber type distribution and cross-sectional area of Type I, I+II or II fibers of soleus muscle in [A], and fast twitch oxidative glycolytic (FOG) or fast twitch glycolytic (FG) fibers of plantaris muscle in [B]. Values are presented as mean ± SE (n=3).

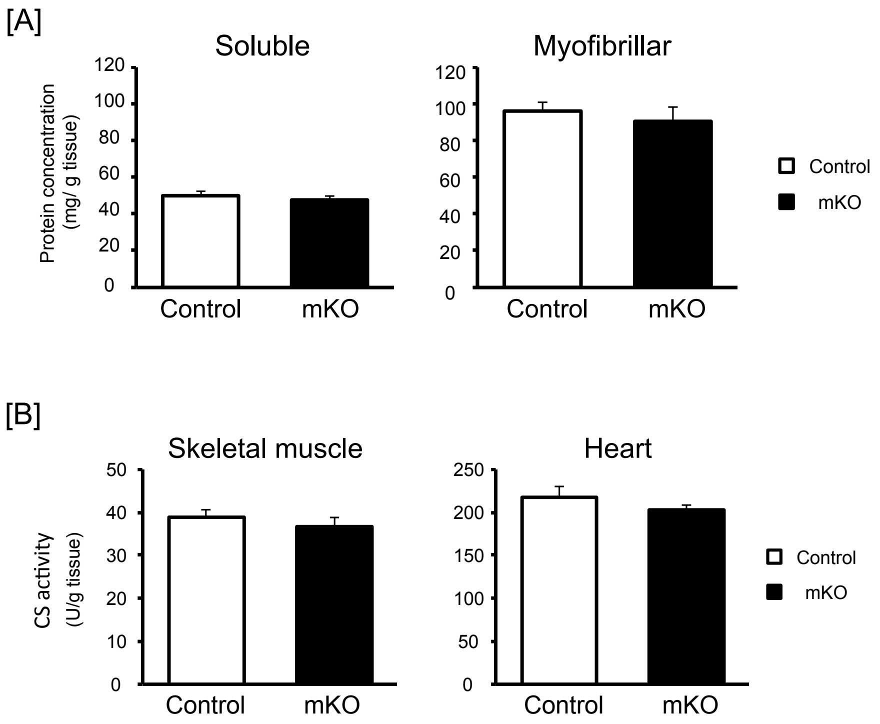

Supplemental Figure S3. Skeletal muscle protein concentrations and mitochondria enzyme activities in control and BDK-mKO mice.

[A] The soluble and myofibrillar protein concentrations in skeletal muscle of 8h-fasted 10-week-old control and BDK-mKO mice fed CE-2 (25% protein). The protein concentrations are expressed as mg/g of tissue weight for skeletal muscle (gastrocnemius and plantaris muscle). **[B]** The activity of citrate synthase of skeletal muscle and heart in 10-week-old control and BDK-mKO mice fed CE-2. Values are presented as mean ± SE (n=10-12).

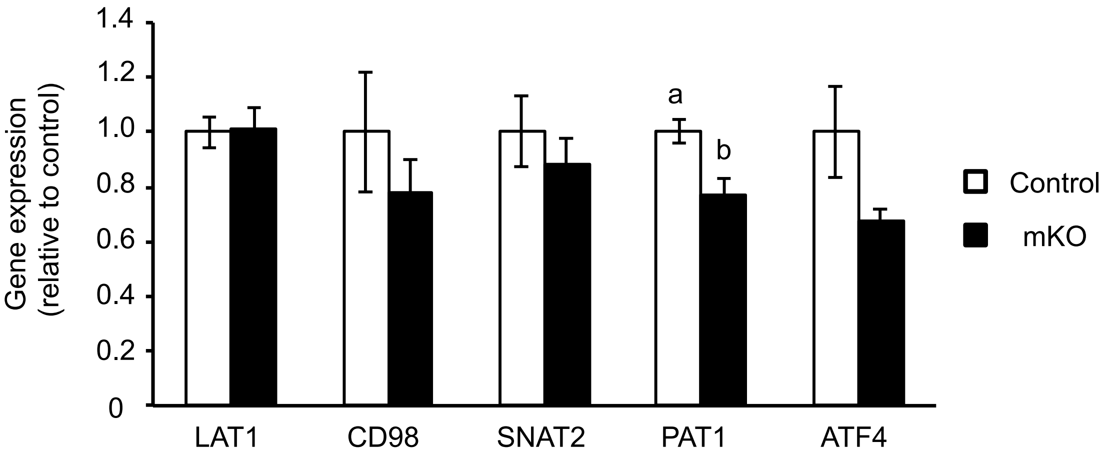

Supplemental Figure S4. The expression of mRNA encoding amino acid transporters in skeletal muscle from control and BDK-mKO mice.

The gene expression of system L amino acid transporters (LAT1 and CD98), system A amino acid transporter (SNAT2), proton-assisted amino acid transporter (PAT1) and ATF4 in skeletal muscle of 8h-fasted BDK-mKO mice fed CE-2 (25% protein) are expressed relative to those from control mice. Values are presented as mean ± SE (n=4). Means without common letters are significantly different (P<0.05 by Student's t test).

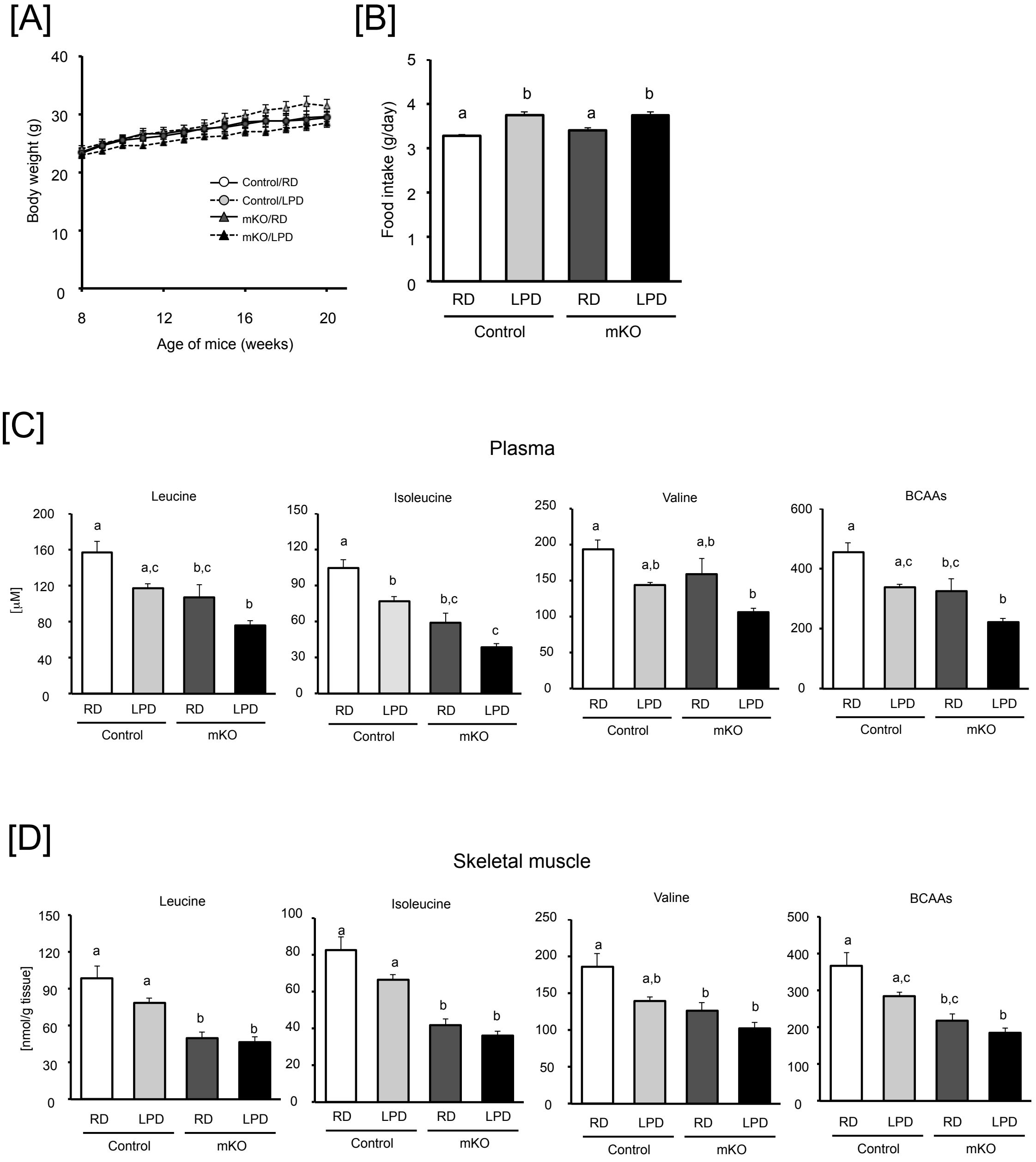

Supplemental Figure S5. Growth curves, food intake and BCAA concentrations of control and BDK-mKO mice fed RD or LPD for 12 weeks.

Growth curves **[A]**, food intake **[B]**, concentrations of leucine, isoleucine, valine and BCAAs (2h fasted) in plasma **[C]** and skeletal muscle **[D]** of control and mKO mice fed regular protein diet (RD, 20% protein) and low protein diet (LPD, 8% protein). Values are presented as mean ± SE (n=4-7). Means without common letters are significantly different (P<0.05 by the Tukey-Kramer test).

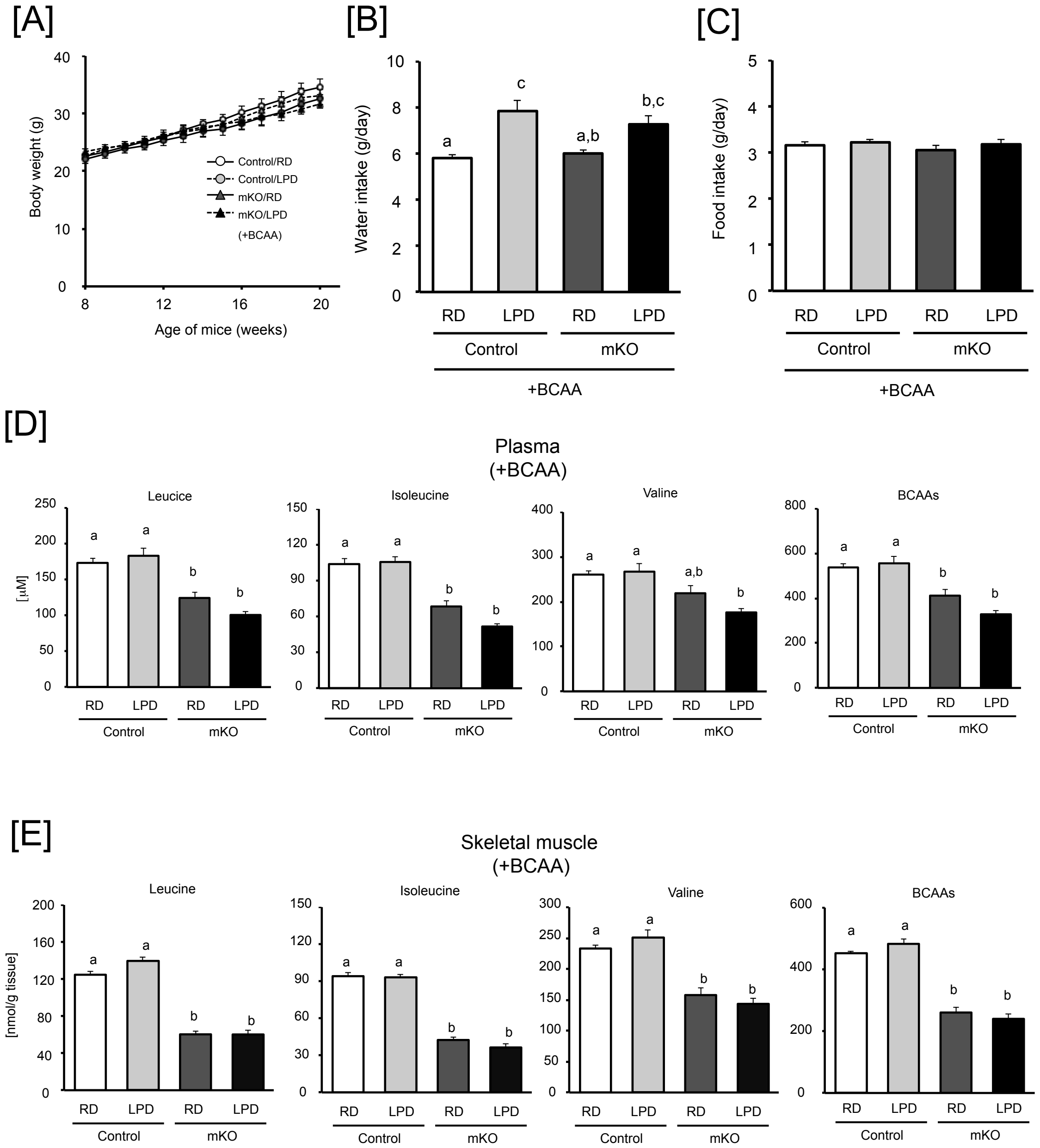

Supplemental Figure S6. Growth curves, food and water intake of control and mKO mice fed RD or LPD for 12 weeks with BCAA supplemented water.

Growth curves **[A]**, water intake **[B]**, food intake **[C]** and concentrations of leucine, isoleucine, valine and BCAAs (2h-fasted) in plasma **[D]** and skeletal muscle **[E]** of control and mKO mice fed RD (20% protein) or LPD (8% protein) with BCAA supplemented water. Values are presented as mean ± SE (n=5-8). Means without common letters are significantly different (P<0.05 by the Tukey-Kramer test).

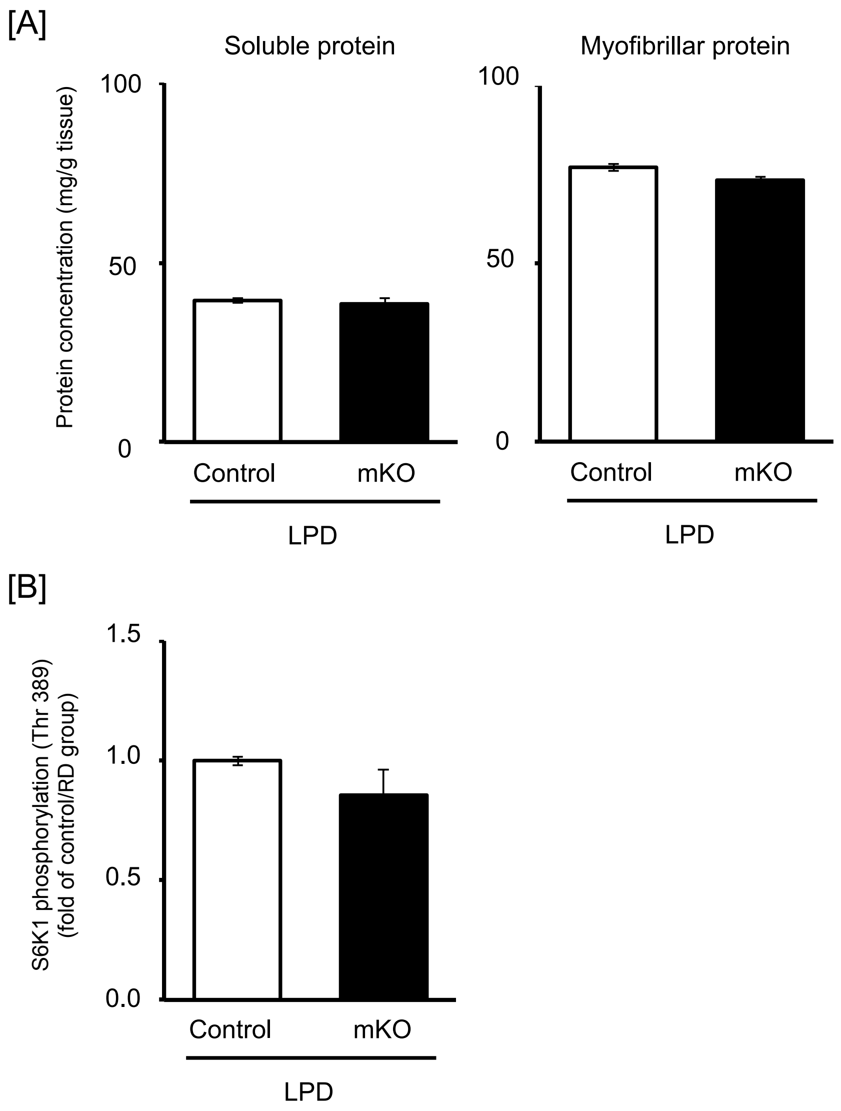

Supplemental Figure S7. Skeletal muscle protein concentrations and mTORC1 activity in control and BDK-mKO mice fed RD or LPD for 4 weeks.

[A] Soluble and myofibrillar protein concentrations in skeletal muscle of 2 h-fasted control and BDK-mKO mice fed RD (20% protein) or LPD (8% protein) for 4 weeks. The protein concentrations are expressed as mg/g of tissue weight for skeletal muscle (gastrocnemius and plantaris muscle). **[B]** The activity of mTORC1 in skeletal muscle of 2 h-fasted control and BDK-mKO mice fed RD or LPD for 4 weeks. The phosphorylated levels of S6K1 and 4E-BP1 are expressed as in Fig. 2. The data are presented relative to the mean values of the control/RD group. Typical images of western blots are shown above each bar. Values are presented as mean ± SE (n=4-7).

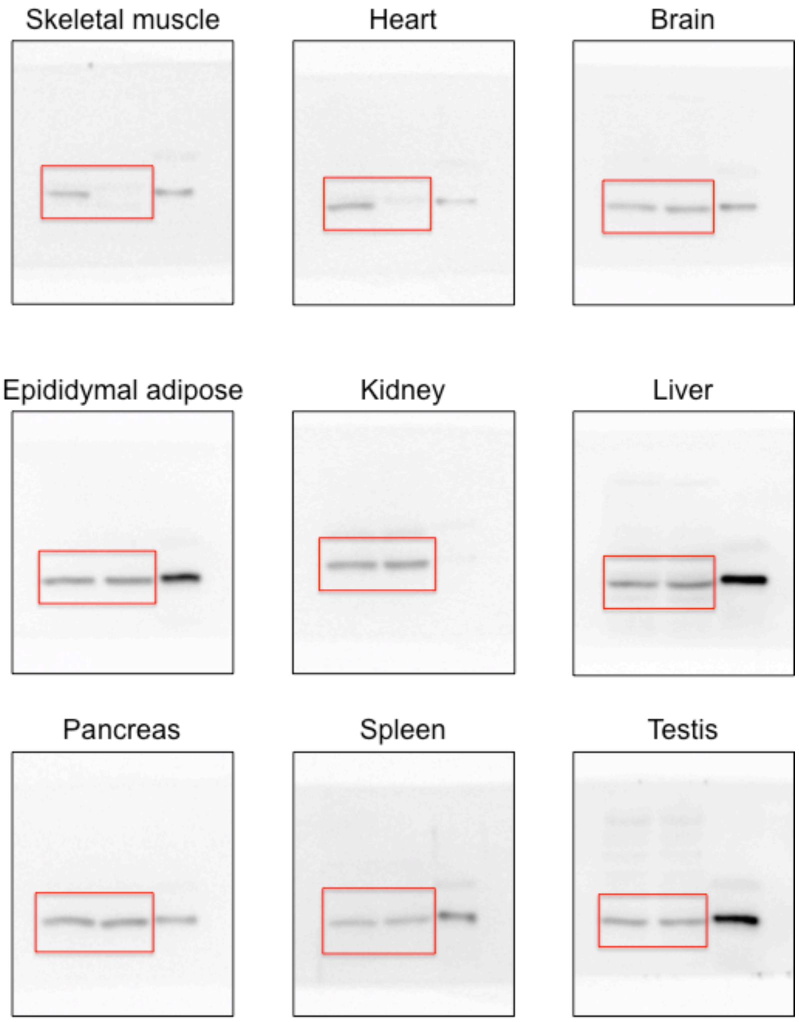

Supplementary Figure S8. Full-length gels/blots of Figure 1 [A].

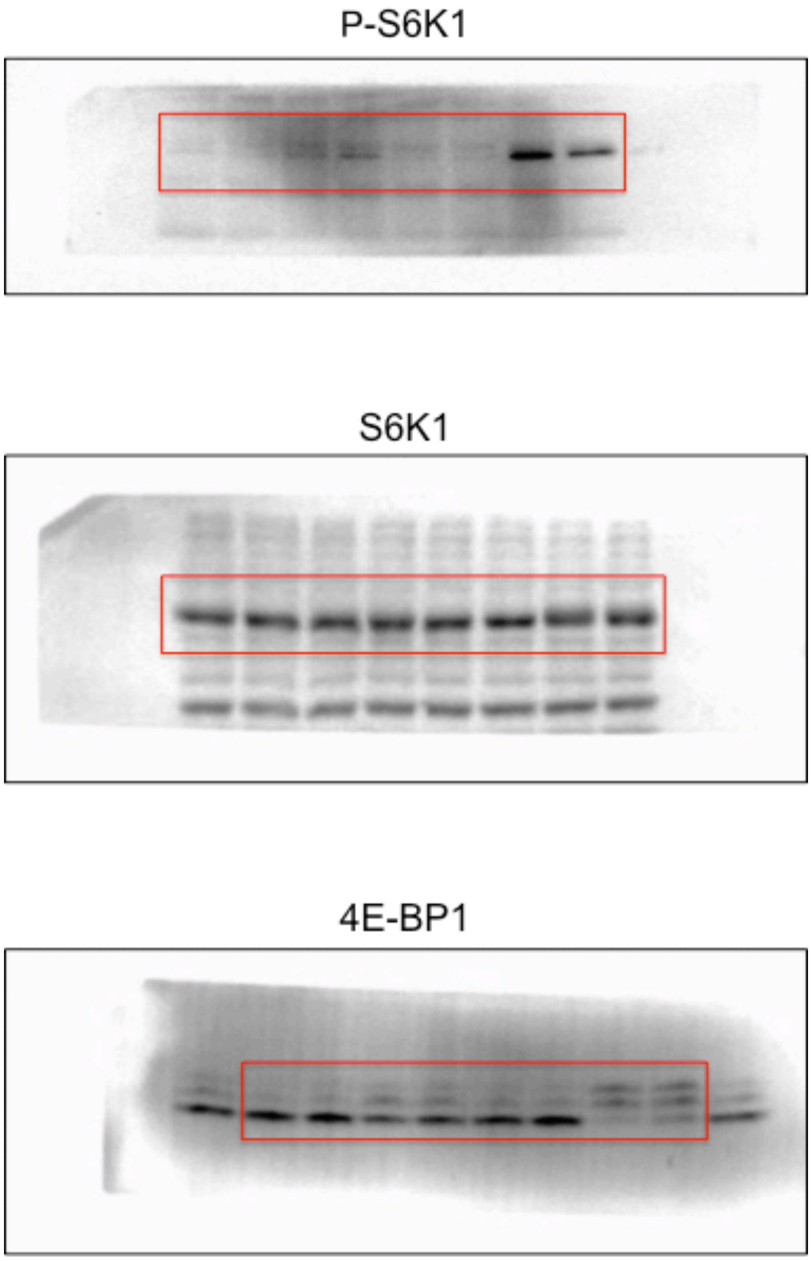

Supplementary Figure S9. Full-length gels/blots of Figure 2.

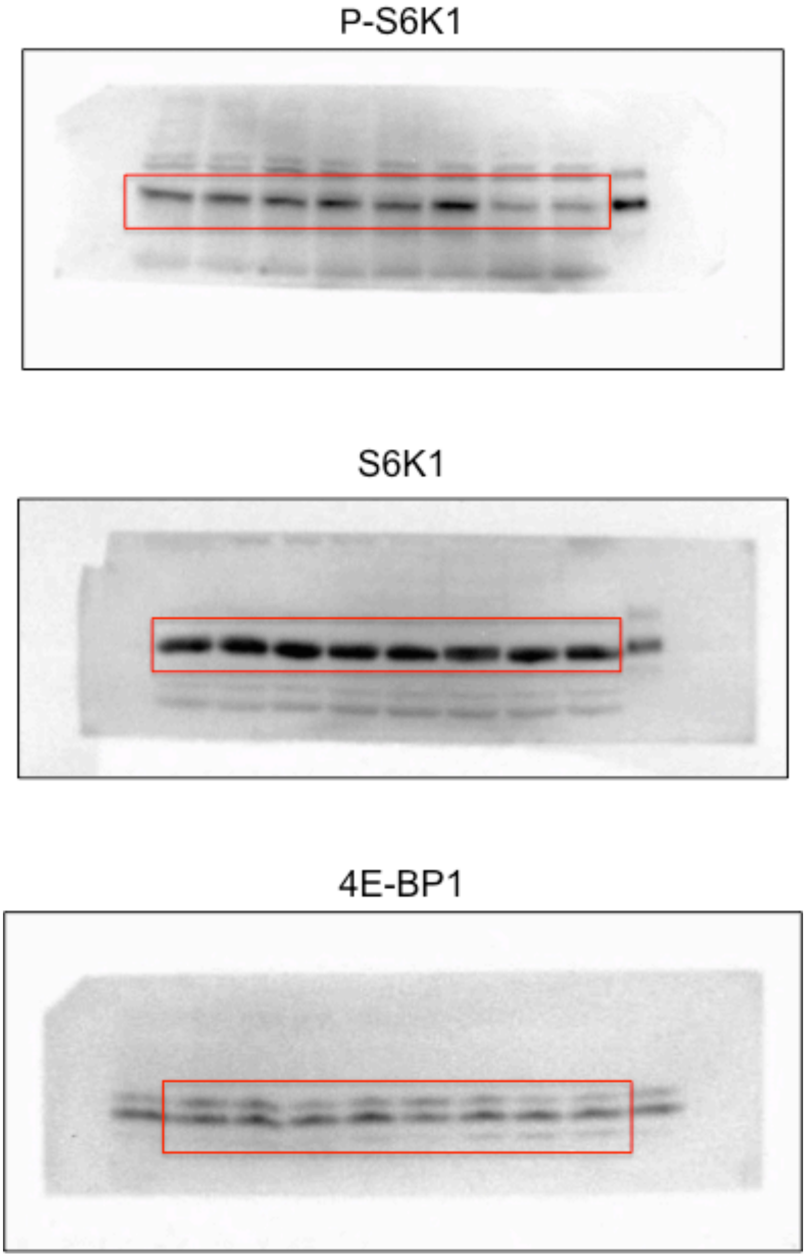

Supplementary Figure S10. Full-length gels/blots of Figure 3[A] and [B].

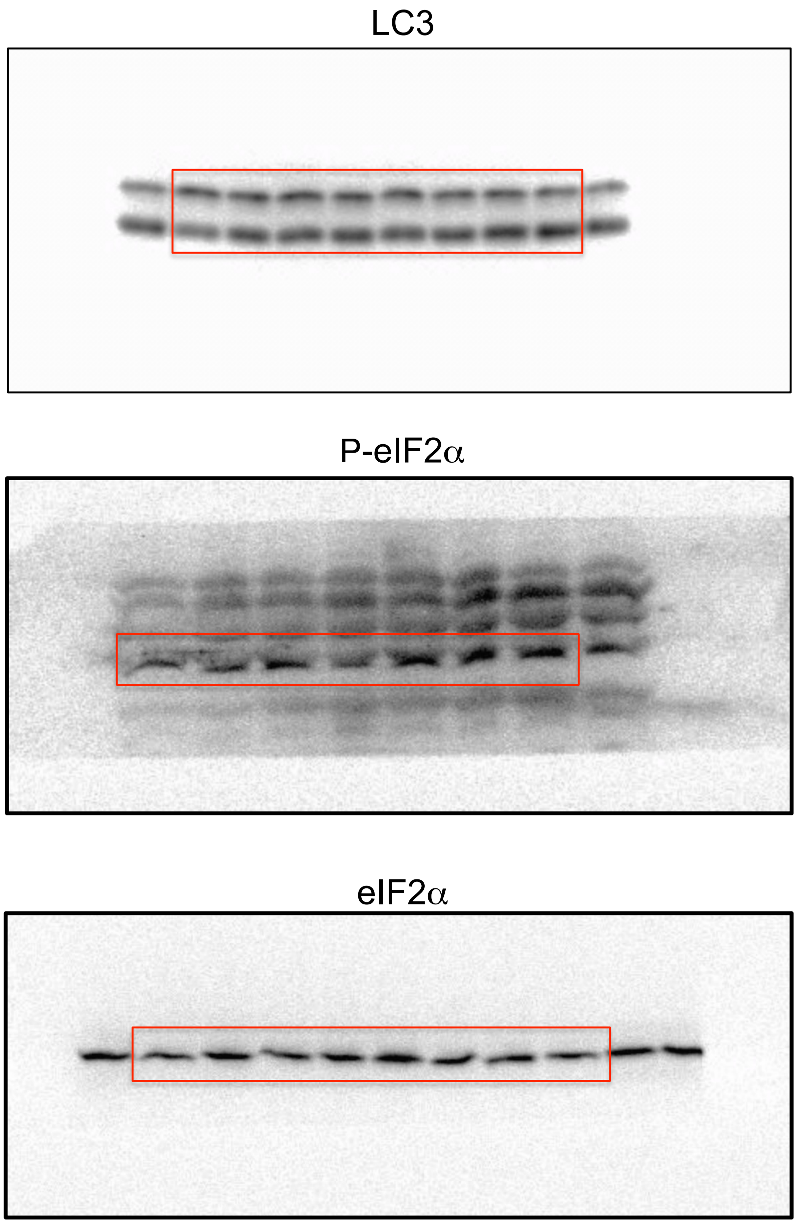

Supplementary Figure S11. Full-length gels/blots of Figure 3[C] and [D].

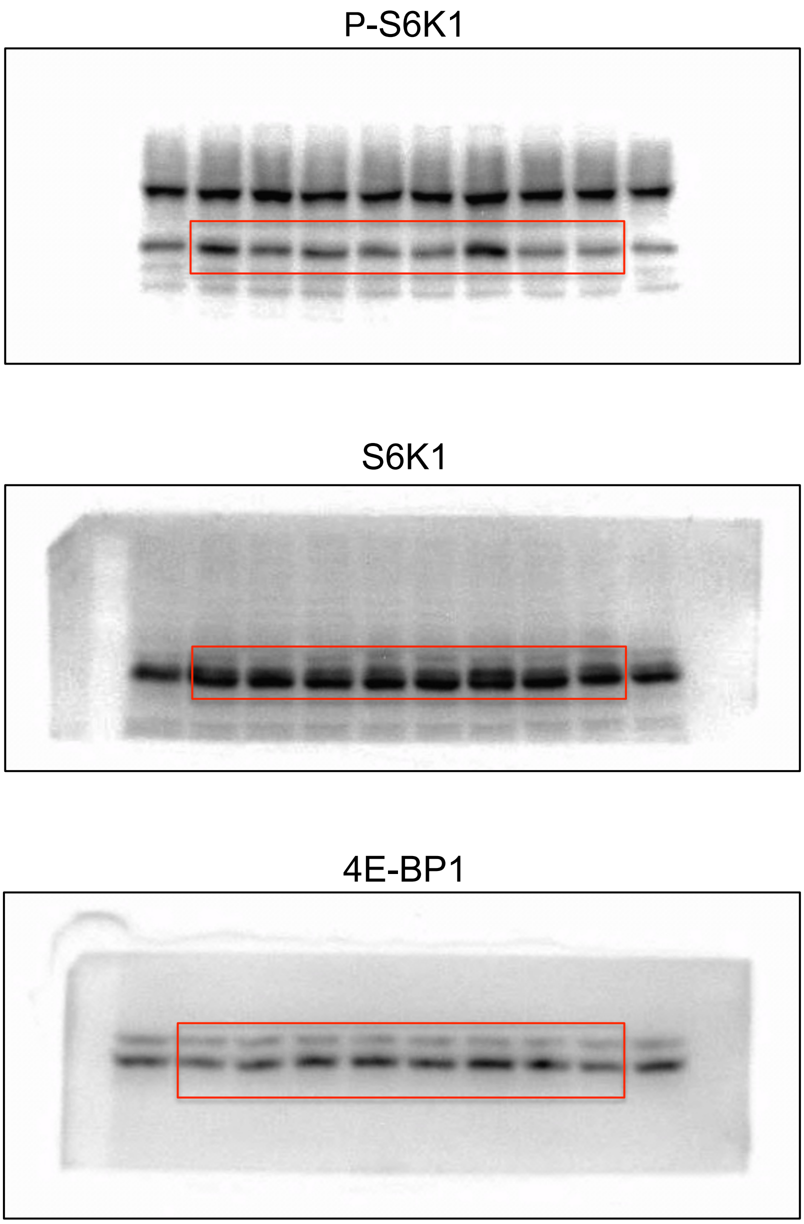

Supplementary Figure S12. Full-length gels/blots of Figure 4.

Supplemental Table S1. List of genes up- or down-regulated in BDK-mKO mice by microarray. Listed genes were up-regulated more than 2-fold or down-regulated less than half in extensor digitorum longus (EDL) and soleus from BDK-mKO mice compared to control mice fed CE-2 (25% protein) at freely-fed state.

EDL

GeneSymbol	Description	mKO/Control
3300002I08Rik	Mus musculus RIKEN cDNA 3300002I08 gene (3300002I08Rik), mRNA [NM_027017]	8.77
Acyp2	Mus musculus acylphosphatase 2, muscle type (Acyp2), mRNA [NM_029344]	2.24
Angptl4	Mus musculus angiopoietin-like 4 (Angptl4), mRNA [NM_020581]	0.39
Egr1	Mus musculus early growth response 1 (Egr1), mRNA [NM_007913]	0.33
Zfp36l3	Mus musculus zinc finger protein 36, C3H type-like 3 (Zfp36l3), mRNA [NM_001009549]	0.29
Hat1	Mus musculus histone aminotransferase 1 (Hat1), mRNA [NM_026115]	0.22
Bckdk	Mus musculus branched chain ketoacid dehydrogenase kinase (Bckdk), mRNA [NM_009739]	0.03

Soleus

GeneSymbol	Description	mKO/Control
3300002I08Rik	Mus musculus RIKEN cDNA 3300002I08 gene (3300002I08Rik), mRNA [NM_027017]	3.75
Angptl4	Mus musculus angiopoietin-like 4 (Angptl4), mRNA [NM_020581]	0.40
Egr1	Mus musculus early growth response 1 (Egr1), mRNA [NM_007913]	0.35
Bckdk	Mus musculus branched chain ketoacid dehydrogenase kinase (Bckdk), mRNA [NM_009739]	0.07

Supplemental Table S2. The concentrations of leucine, isoleucine, valine and total BCAAs in the plasma and skeletal muscle of control and BDK-mKO mice fed RD (20% protein) at 1 h after bolus ingestion of water or BCAAs.

The concentrations are expressed as μM for plasma or nmol/g tissue for skeletal muscle. Values are presented as mean ± SE (n=4-6). Means without common letters are significantly different ($P<0.05$ by the Tukey-Kramer test).

Plasma

	Control/water	Control/BCAAs	BDK-mKO/water	BDK-mKO/BCAAs
Leucine	183 ± 19 [a]	1433 ± 61 [b]	119 ± 8 [a]	1203 ± 74 [c]
Isoleucine	117 ± 11 [a]	720 ± 32 [b]	64 ± 5 [a]	551 ± 31 [c]
Valine	194 ± 19 [a]	1674 ± 41 [b]	148 ± 10 [a]	1418 ± 62 [c]
BCAAs	494 ± 48 [a]	3827 ± 128 [b]	330 ± 23 [a]	3172 ± 166 [c]

Skeletal muscle

	Control/water	Control/BCAAs	BDK-mKO/water	BDK-mKO/BCAAs
Leucine	159 ± 28 [a]	1122 ± 37 [b]	98 ± 6 [a]	899 ± 37 [c]
Isoleucine	125 ± 19 [a]	589 ± 20 [b]	73 ± 4 [a]	450 ± 19 [c]
Valine	183 ± 34 [a]	1234 ± 59 [b]	141 ± 9 [a]	1073 ± 34 [b]
BCAAs	467 ± 81 [a]	2944 ± 116 [b]	312 ± 19 [a]	2422 ± 90 [b]

Supplemental Table S3. Skeletal muscle weights of control and BDK-mKO mice fed RD (20% protein) or LPD (8% protein) for 12 weeks.

Weights of the Muscle R and Muscle L correspond to the sum of the gastrocnemius, plantaris and soleus, and the sum of gastrocnemius and plantaris, respectively. Values are presented as mean ± SE (n=4-7).

Relative weight [g/100 g BW]

	Control/RD	Control/LPD	BDK-mKO/RD	BDK-mKO/LPD
Muscle L	0.54 ± 0.03	0.50 ± 0.01	0.55 ± 0.01	0.56 ± 0.02
Muscle R	0.58 ± 0.01	0.55 ± 0.02	0.59 ± 0.01	0.58 ± 0.01
Soleus	0.03 ± 0.00	0.03 ± 0.00	0.03 ± 0.00	0.03 ± 0.00

Supplemental Table S4 Skeletal muscle weights of control and BDK-mKO mice fed RD (20% protein) or LPD (8% protein) for 12 weeks with BCAA supplemented water.

Weights of the Muscle R and Muscle L correspond to the sum of the gastrocnemius, plantaris and soleus, and the sum of gastrocnemius and plantaris, respectively. Values are presented as mean ± SE (n=5-8).

Relative weight [g/100 g BW]

	Control/RD	Control/LPD	BDK-mKO/RD	BDK-mKO/LPD
Muscle L	0.47 ± 0.01	0.49 ± 0.02	0.50 ± 0.02	0.53 ± 0.02
Muscle R	0.51 ± 0.01	0.53 ± 0.02	0.53 ± 0.02	0.56 ± 0.02
Soleus	0.03 ± 0.00	0.03 ± 0.00	0.03 ± 0.00	0.03 ± 0.00

Supplemental Table S5. The concentrations of amino acids other than BCAAs in the plasma and skeletal muscle of 2 h-fasted control and BDK-mKO mice fed RD (20% protein) or LPD (8% protein) for 12 weeks.

The concentrations are expressed as μM for plasma or nmol/g tissue for skeletal muscle. Values are presented as mean ± SE (n=5-8). Means without common letters are significantly different (P<0.05 by the Tukey-Kramer test).

Plasma

	Control/RD	Control/LPD	BDK-mKO/RD	BDK-mKO/LPD
Alanine	465 ± 74	527 ± 46	521 ± 50	582 ± 31
Arginine	90 ± 6	102 ± 2	85 ± 8	95 ± 5
Asparagine	103 ± 15	126 ± 7	107 ± 10	120 ± 5
Aspartic acid	21 ± 3[a,b]	21 ± 1[a,b]	19 ± 2[a]	28 ± 2[b]
Glutamic acid	55 ± 7	44 ± 6	54 ± 8	51 ± 5
Glutamine	515 ± 27[a,b]	518 ± 15[a,b]	486 ± 21[a]	590 ± 16[b]
Glycine	255 ± 15	273 ± 15	257 ± 15	241 ± 8
Histidine	59 ± 7	42 ± 2	56 ± 4	55 ± 6
Lysine	286 ± 24	257 ± 7	315 ± 30	292 ± 9
Methionine	84 ± 12	70 ± 7	81 ± 12	63 ± 8
Phenylalanine	74 ± 7	65 ± 3	71 ± 3	61 ± 4
Serine	150 ± 15	173 ± 9	149 ± 15	184 ± 6
Threonine	208 ± 20	236 ± 13	213 ± 25	267 ± 8
Tyrosine	120 ± 10	121 ± 8	126 ± 9	124 ± 7

Skeletal muscle

	Control/RD	Control/LPD	BDK-mKO/RD	BDK-mKO/LPD
Alanine	2589 ± 232 [a,c]	2382 ± 127 [a]	3035 ± 126 [c]	3386 ± 103 [b]
Arginine	240 ± 27 [a]	260 ± 15 [a]	287 ± 37 [a,b]	392 ± 38 [b]
Asparagine	180 ± 17	191 ± 12	191 ± 13	225 ± 11
Aspartic acid	881 ± 45 [a,b]	772 ± 30 [a]	882 ± 58 [a,b]	991 ± 35 [b]
Glutamic acid	648 ± 45 [a,b]	510 ± 51 [a]	697 ± 61 [a,b]	780 ± 60 [b]
Glutamine	1530 ± 116 [a]	1392 ± 88 [a]	1557 ± 97 [a]	2096 ± 73 [b]
Glycine	2277 ± 188	2674 ± 180	2295 ± 128	2640 ± 101
Histidine	121 ± 32 [a]	148 ± 6 [a]	173 ± 8 [a]	218 ± 8 [b]
Lysine	701 ± 202	814 ± 66	1294 ± 216	1451 ± 168
Methionine	70 ± 7	62 ± 3	70 ± 6	61 ± 2
Phenylalanine	50 ± 8	42 ± 2	46 ± 4	44 ± 3
Serine	327 ± 37 [a]	380 ± 23 [a]	349 ± 23 [a]	517 ± 27 [b]
Threonine	387 ± 51 [a]	408 ± 22 [a]	429 ± 38 [a]	627 ± 35 [b]
Tyrosine	99 ± 10	101 ± 9	115 ± 5	121 ± 3

Supplemental Table S6. Experimental diets

**Name**	**Regular Diet (RD)**		**Low Protein Diet (LPD)**	
**Product #**	**D12450J**		**D06011002**	
	**g%**	**kcal%**	**g%**	**kcal%**
Protein	19	20	8	8
Carbohydrate	67	70	79	82
Fat	4	10	4	10
**Total**		**100**		**100**
**kcal/g**	**3.8**		**3.8**	
**Ingredient**	**g**	**kcal**	**g**	**kcal**
Casein	200	800	79.8	319
L-Cystine	3	12	1.2	5
Corn Starch	506.2	2025	628.2	2513
Maltodextrin	125	500	125	500
Sucrose	68.8	275	68.8	275
Cellulose, BW200	50	0	50	0
Soybean Oil	25	225	25	225
Lard	20	180	20	180
Mineral Mix S10026	10	0	10	0
DiCalcium Phosphate	13	0	13	0
Calcium Carbonate	5.5	0	5.5	0
Potassium Citrate, 1 H_2O	16.5	0	16.5	0
Vitamin Mix V10001	10	40	10	40
Choline Bitartrate	2	0	2	0
**Total**	**1055**	**4057**	**1055**	**4057**
